# Changes in the Anatomic and Microscopic Structure and the Expression of HIF-1α and VEGF of the Yak Heart with Aging and Hypoxia

**DOI:** 10.1371/journal.pone.0149947

**Published:** 2016-02-25

**Authors:** Yanyu He, Sijiu Yu, Junwei Hu, Yan Cui, Penggang Liu

**Affiliations:** College of Veterinary Medicine, Gansu Agricultural University, Lanzhou 730070, Gansu, China; Northwestern University, UNITED STATES

## Abstract

The study aimed to identify the changes of anatomic and microscopic structure and the expression and localization of hypoxia-inducible factor (HIF)-1α and vascular endothelial growth factor (VEGF) in the myocardium and coronary artery of the yak heart adapted to chronic hypoxia with aging. Thirty-two yaks (1 day, 6 months, 1 year, 2 years, and 5 year old) were included, and immunoelectronmicroscopy, immunohistochemistry, and enzyme-linked immunosorbent assay (ELISA) were used. Right ventricular hypertrophy was not present in yaks with aging. There was no intima thickening phenomenon in the coronary artery. The ultrastructure of myofibrils, mitochondria, and collagen fibers and the diameter and quantity of collagen changed significantly with aging. The enzymatic activity of complexes I, II, and V increased with age. Immunogold labeling showed the localization of HIF-1α protein in the cytoplasm and nuclei of endothelial cells and cytoplasm of cardiac muscle cells, and VEGF protein in the nuclei and perinuclei areas of smooth muscle cells of coronary artery, and in the cytoplasm and nuclei of endothelial cells. ELISA results showed that HIF-1α secretion significantly increased in the myocardium and coronary artery from an age of 1 day to 2 years of yaks and decreased in old yaks. However, VEGF protein always increased with aging. The findings of this study suggest that 6 months is a key age of yak before which there are some adaptive changes to deal with low-oxygen environment, and there is a maturation of the yak heart from the age of 6 months to 2 years.

## Introduction

The Tibetan highlands are one of the most extreme environments inhabited by humans and animals [1,2]. Here we investigated how the Tibetan Yak obtained genetic features of high-altitude hypoxia adaption at the physiological, morphological, and genetic levels. The adaptability of yaks to the harsh environment of the high plateau is especially interesting because exposure of other cattle to low oxygen levels cause Bovine high-moutain or Brisket disease characterized by pulmonary hypertension and right ventricular hypertrophy. Yaks living on the Qinghai Tibet Plateau demonstrate less sensitivity and good adaptability to hypoxia [3,4]. Undoubtedly, the yak heart plays an important role in the high-altitude hypoxia adaptation; therefore, the study of its morphology is meaningful.

Previously, many researchers have investigated specific facets of the anatomic structure [5–8] and microscopic structure of heart in other animals [9–12]. However, there has been no detailed report on the anatomic and microscopic structure of the yak heart, which does not transition to “normal”oxygen levels (21% O_2_) after birth.

Hypoxia contributes significantly to the pathophysiology of human heart diseases [13]. Hypoxia-inducible factor (HIF-1α) is a major regulator for oxygen homeostasis [14] and plays a role in tissue remodeling during physiological adaption to moderate levels of systemic hypoxia [15,16]. Hypoxia is also one of the potent stimulators for vascular endothelial growth factor (VEGF) expression [17] and the HIF-1αpathway is one of the important mechanisms by which VEGF production is increased during hypoxia or ischemia [18,19]. Following hypoxia, HIF-1α is stabilized and is targeted to the nucleus where it bindsto the hypoxia response elements in the 5’flanking region of the VEGF gene. VEGF expression then plays an important role in the vascular changes during chronic hypoxia [15]. Numerous reports on the effects of acute hypoxia on the heart are available, but effects of chronic hypoxia on heart acclimatization have not been established.

Previously, it was found that the microvasculature and conduction system of the yak heart has unique characteristics [20,21], suggesting that the vasculature of the yak heart constructs an adaptive structure to cope with the low-oxygen situation. However, there is a little information on the levels of protein expression of HIF-1α and VEGF following the birth of yaks, and during their maturation at high altitude. Hence, our objectives was to demonstrate the changes of anatomic and microscopic structure of the yak heart, and the expression and localization of HIF-1α and VEGF in the myocardium and coronary artery of the yak heart adapting to chronic hypoxia. These results would be useful to help us understand how the heart adapts to life in a low oxygen environment and could provide insight into the adaptations of the human heart at high altitudes.

## Materials and Methods

### Yak

A total of 32 yaks (16 females, 16 males) of 1 day, 6 months, 1 year, 2 years, and 5 years in age were included in this study. All animals were kept under the conditions same as the natural condition. Yaks were purchased from small holders in Datong County of Qinghai Province (China). No apparent diseases were found before they were sampled. In this study, experimental animals were all handled in the mercy way according to the Animal Ethics Procedures and Guidelines of the People’s Republic of China, and the study was approved by the Animal Ethics Committee of Gansu Agricultural University. Yaks were permitted as experimental animals by the owner and were killed by exsanguinated via abdominal aorta in slaughter house after anesthesia.

### Sampling for transmission electron microscopy

The myocardium of the right and left ventricles and rami anterior descendants of the coronary artery were selected for this study. Two blocks (1 mm^3^ each) of myocardium and rami anterior descendants were cut with a razor blade to make transverse and longitudinal ultrathin sections, and the sections were fixed in 3% glutaraldehyde at 4°C for 12 h. Then the samples were post-fixed with 1% osmium tetroxide (OSO_4_) in phosphate-buffered saline (PBS) for 2 h at 4°C, dehydrated in an increasing ethanol series, and embedded in SPION-PON 812 (SPI-CHEM, Lot NO: 1100107, westchester, PA, made in USA). Ultrathin sections of myocardium and rami anterior descendants 70 nm thickness were prepared with an ultratome (Nova, LKB, Bromma, Sweden), stained with uranyl acetate and lead citrate, and examined under a JEM-1230 electron microscope (JEOL Ltd., Tokyo, Japan).

### Immunochemistry for electron microscopy

The myocardium of the right and left ventricles and rami anterior descendants of the coronary artery were selected for this study. Sections of 50 μm thickness were prepared with a vibratome (VT 1000S; Leica, Heidelberger, Nussloch, Germany) for HIF-1α and VEGF immunohistochemical labeling. Sections were immersed in PBS containing 5% bovine serum albumin and 5% normal goat serum for 4 h to block nonspecific immunoreactivity. HIF-1α and VEGF were detected with the immunogold-silver staining method. Sections were incubated overnight with mouse anti-HIF-1α monoclonal antibody and rabbit anti-VEGF polyclonal antibody (1:200, Abcam, Hong Kong, ab463 and ab53465), and then were incubated with goat-anti-mouse and goat-anti-rabbit IgG conjugated to 1.4 nm gold grains (1:100, Nanoprobes, NY, USA) overnight, respectively. Silver-enhancement was carried out in the dark with the HQ Silver Enhancement Assay Kit (Nanoprobes, NY, US) for visualizing HIF-1α and VEGF immunoreactivity. Before and after the sliver enhancement step, sections were rinsed several times with the deionized water. Immunolabeled sections were fixed with 0.5% OSO_4_ in 0.1 M phosphate buffer for 1 h, dehydrated in graded ethanol series, then in propylene oxide, and finally flat-embedded in SPION-PON 812 (SPI-CHEM, Lot NO: 1100107, west chester, PA, made in USA). After polymerization, sections were examined under a light microscope. The stratum radiatum HIF-1α and VEGF subregion was selected and trimmed under a stereomicroscope and mounted onto blank resin stubs. Ultrathin sections were cut with an ultratome (Nova, LKB, Bromma, Sweden) and mounted on mesh grids (six to eight sections/grid). The ultrathin sections were counterstained with uranylacetate and lead citrate and observed under a JEM-1230 electron microscope (JEOL Ltd., Tokyo, Japan).

### Mitochondrial enzymatic activity

The blocks of ventricularwall from the left and right heart were sampled (six yaks each group). Mitochondria were isolated by GenMed animal tissue mitochondrial isolation kit (Lot No. 2-005116-12). According to the kit instructions, we weighed the same weight (0.1 g) for each sample to carry on the analysis. The enzymatic activity of complexes I, II, III, IV, and V was evaluated using GenMed animal mitochondrial respiratory chain complexes I, II, III, IV, and V activity quantitative determination kits (Lot No. 1-423517-14; 1-256978-73; 1-423517-14; 1-45017-10; 1-423517-10). All these reagent kits were purchased from GenMed Scientifics, Shanghai, China. The enzymatic activity of mitochondria was measured by the spectrophotometer (UV-2550, SHIMADZU, Japan).

### Light microscopy

The left circumflex of coronary artery was selected for this study. The left circumflex of coronary artery were cut to 1 mm^2^ with a razor blade and fixed with 4% formalin in PBS at 4°C for 24 h. After conventional paraffin wax embeding, 5 μm serial sections were cut, and the sectons were stained with Ver-hoeffes Van Gession (VVG) (elastic fiber—black, collagen fiber—red and smooth muscle—yellow or red) and Massion trichrome (elastic fiber—pink, collagen fiber—blue and smooth muscle cell—red) to observe the elastic tissue, collagen fiber and smooth muscle. All sections were reviewed and photographed using an Olympus DP71 microscope.

### Localization of HIF-1α and VEGF in myocardium and coronary artery by immunochemistry

Tissue were harvested and fixed with 4% formalin in PBS at 4°C for 24 h. The fixed samples were dehydrated, embedded with paraffin, and cut into 5-μm-thick sections. The sections were rehydrated, blocked with 5% goat serum, and incubated overnight at 4°C with the primary antibody of mouse anti-HIF-1α monoclonal antibody and rabbit anti-VEGF polyclonal antibody (1:200, Abcam, Hong Kong, ab463 and ab53465). The sections were then incubated with the secondary antibody. The labeled samples were then counterstained with 3–3'-diaminobenzidine.

### Detection of HIF-1α and VEGF by ELISA

HIF-1α and VEGF protein involved in myocardium and coronary artery were directly measured by ELISA kit according to the manufacturer’s instructions (Bovine VEGF ELISA Kit, BO60014, Bio-Swamp, Wuhan Beinglay Biotech Co., LTD, wuhan, China; Bovine HIF-1α ELISA Kit, BO60029, Bio-Swamp, Wuhan Beinglay Biotech Co., LTD, wuhan, China). The PBS solution used for grinding tissue was analyzed as a control. The absorbance was measured at 450 nm. The concentration of HIF-1α and VEGF were calibrated with the HIF-1α and VEGF standard curve.

### Quantification of collagen

For light microscopy, segments of the myocardium tissue were fixed in Bouin’s solution for 48h, embedded in paraffin and cut tangentially at 5μm thickness. Sections were stained in the Picrosirius solution [22] and studied with polarization microscopy. When studied by this method, the evidence presented suggest that tissues containing collagen type I show thick, strongly birefringent yellow or red fibers, while collagen type III appears as thin, pale, greenish fibers [23].

### Measurements and statistical analysis

The measurements of external diameters of hearts were done according to the norm of anatomy and high-altitude medicine. The perimeter of heart was measured along the coronary sulcus. Heart length was the vertical distance from the apex to the junction of right coronary sulcus and the right longitudinal furrow. The thickness of the ventricular wall was measured from coronary artery, central, and apex of heart.

For quantifying the density of mitochondria (100 microscopic field each yak, and four yaks each group), the images of ultrathin sections of the longitudinal tissue of ventricular myocardium were taken with transmission electron microscope (TEM) and measured with Image Pro-plus 6.0 (Media Cybernetics, Inc, MD, US). For measuring the diameter of collagen (the-100-root-of collagen fibers each yak, four yaks each group), transverse ultrathin sections were used. For quantification of the collagen type Ⅰand III (100 microscopic field each yak, and four yaks each group), the images of tangentially cut of ventricular myocardium were taken with polarization microscopy and measured with Image Pro-plus 6.0. The data were expressed as the mean ±standard deviation and were analyzed by one-way ANOVA using the SPSS software (version 17.0). A p value of < 0.05 was considered statistically significant.

## Results

### The changes of anatomical structure

Weight, perimeter, and length of the yak heart (Table 1), and thickness of the ventricular wall (Table 2) increased with aging of yaks. Table 2 revealed that the right hypertrophy was not presented in yaks, and the yak heart belonged to the left dominant pattern.

**Table 1:** The weight of heart and yak, and the length of heart perimeter.

Age	Weight of yak (kg)	Weight of heart (kg)	Heart perimeter (cm)	Heart length (cm)	TM length (cm)	TM diameter (cm)
1 day	10.74±1.71a	0.13±0.03a	15.74±0.93a	6.21±0.66a	1.46±0.17a	0.183±0.01a
6 months	25.80±1.59b	0.25±0.02b	23.21±1.93b	8.82±1.32a	1.90±0.25a	0.236±0.06b
1 year	40.47±8.01c	0.32±0.05b	26.65±2.11b	10.98±1.27a	2.00±0.18b	0.340±0.88b
2 years	87.09±10.73d	1.13±0.06c	31.94±2.49c	14.22±1.90b	2.21±0.59b	0.769±1.02c
5 years	107.33±20.49e	1.57±0.10d	35.50±2.71c	15.15±2.00b	2.61±0.30c	0.799±0.99c

Note: Values are means ± SD. One hundred hearts of yaks in each age group were measured. In the same column, different letters a, b, c, d and e represent the difference was significant (p < 0.05), the same letter represent that the difference was not significant in the same column (p > 0.05). TM:Transverse muscle.

**Table 2:** Thickness of the ventricular wall of heart in yak. Unit: cm.

Position	Left ventricle			Right ventricle			Interventricular septum		
	Coronary	Central	Apex	Coronary	Central	Apex	Coronary	Central	Apex
1 day	0.90±0.42a	1.03±0.18a	0.96±0.28a	0.58±0.20a	0.66±0.15a	0.45±0.23a	0.87±0.45a	1.21±0.34a	0.44±0.24a
6 month	1.72±0.27b	1.97±0.21b	1.21±0.19b	0.65±0.17b	0.80±0.12b	0.51±0.21a	1.31±0.35b	1.82±0.22b	0.92±0.16a
1 year	1.81±0.35b	2.36±0.51b	1.49±0.38b	0.70±0.55b	0.92±0.20b	0.59±0.24b	1.67±0.40b	2.01±0.39b	1.24±0.48b
2 year	2.08±0.17b	2.98±0.19c	1.87±0.30c	0.94±0.62c	1.11±0.16c	0.69±0.20c	2.13±0.29c	2.69±0.13b	1.90±0.38b
5 year	3.07±1.22c	3.56±0.79c	2.18±0.40c	1.10±0.59c	1.33±0.25c	0.72±0.32c	4.99±1.23d	5.15±0.80c	4.26±0.62c

Note: Values are means ± SD. One hundred hearts of yaks in each age group were measured. Different letters a, b and c represent that the difference was significant in the same column (p < 0.05), the same letter represent that the difference was not significant in the same column (p > 0.05).

### The changes of microscopic structure

**Fig 1:**
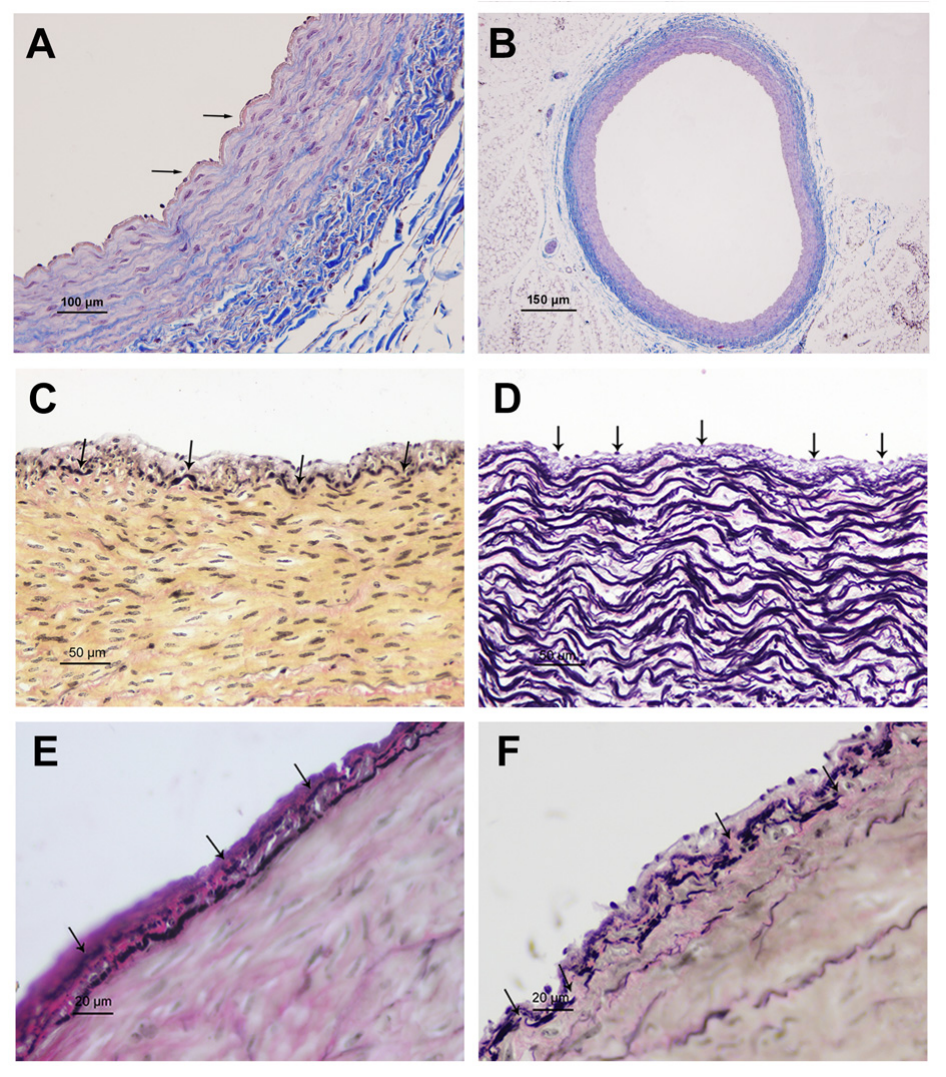
The microscope structures of coronary artery. A: A monolayer of endothelial cell adhered to internal elastic membrane in 1-day old yak (arrowheads) (Massion trichrome). B: An integral structure of the left circumflex of coronary artery in 1-day old yak (Massion trichrome). C: Internal elastic membrane was disrupted in heart of 6-month old yak (arrowheads) (VVG). D: The internal elastic membrane was divided into two layers in 1-year old yak (arrowheads) (VVG). E: Those internal elastic membrane and smooth muscle cells together form Muscle elastic zone in 2-year old yak (arrowheads) (VVG). F: Secondary internal elastic membrane ruptured, or even fall off, and smooth muscle membrane exposed in old yak (arrowheads) (VVG).

Vascular anatomy revealed that the left circumflex coronary artery supplied most of the blood to the heart, and should be considered the dominant branch of the coronary arteries. The thickness of vascular intima and tunicae media vasorum inrami anterior descendants, and left and right circumflex of coronary artery were measured (Table 3). The results showed that vessel wall thickness increased with age, with the most obvious increase presented in rami anterior descendants. A monolayer of endothelial cells adhered to internal elastic membrane in 1-day-old yaks, which had an integral structure and large folding (Fig 1A and 1B). The internal elastic membrane was disrupted, and some longitudinal smooth muscle cells presented near the fracture in the heart of 6-month-old yaks; smooth muscle cells and the internal elastic membrane together form the elastic hyperplasia layer (Fig 1C). In 1-year old yaks, the internal elastic membrane was divided into two layers, with some smooth muscle cells presented between them (Fig 1D). These internal elastic membranes and smooth muscle cells together form a muscle elastic zone. Vascular endothelial cells partially desquamated in 2-year-old yaks (Fig 1E). In old yaks, the secondary internal elastic membrane ruptured, or even fell off, exposing the smooth muscle membrane (Fig 1F).

**Table 3:** Thickness of vascular intima and tunicae media vasorum inrami anterior descendens, left and right circumflex of coronary artery. Unit: μm.

Age	intima			tunicae media		
	AD	LC	RC	AD	LC	RC
1-day	5.01±0.36a	4.45±0.57a	4.31±0.39a	92.26±2.77a	101.98±10.74a	111.14±2.38a
6-month	5.90±0.41a	5.13±0.38a	5.75±0.38a	182.97±7.80b	229.62±6.52b	271.11±3.20b
1-year	7.38±0.59 a	5.77±0.81a	5.91±0.43a	254.24±10.36b	297.11±12.60b	298.34±5.29b
2-year	11.24±1.22b	8.11±0.86b	6.14±0.63b	326.93±13.42c	353.50±10.54c	306.28±1.30c
5-year	12.23±0.93b	8.20±0.72b	6.19±0.55b	361.31±11.28c	358.46±9.19c	307.81±2.05c

**Note:** Values are means ± SD. One hundred hearts of yaks in each age group were measured. Different letters a, b and c represent that the difference was significant in the same column (p < 0.05), the same letter represent that the difference was not significant in the same colum (p > 0.05). RAD: Rami anterior descendens, LC: Left circumflex, RC: Right circumflex.

### Ultrastructural changes in the yak myocardium

**Fig 2:**
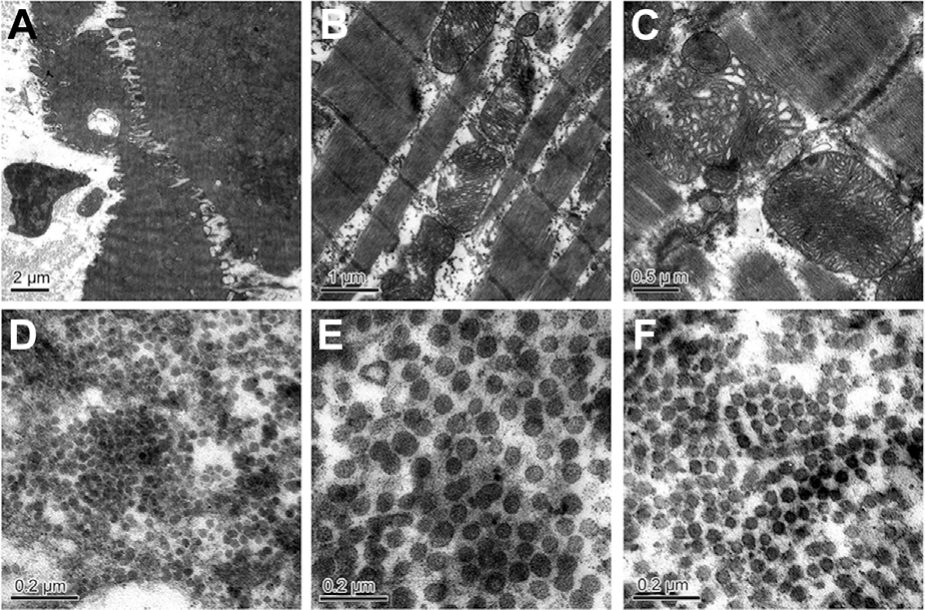
The ultra-structural changes of mitochondria in the yak heart with aging. A: Mitochondria aggregated around the nucleus in 1-day-old yak (arrowheads); B: Mitochondria ridge was uniform and well distributed (arrowheads) in 6-month-old yak. C: Giant mitochondria and the irregular ridge in 5-year-old yak (arrowheads). D, E, F: Collagen fibers in the 1-day-old, 6-month-old, and 2-year-old yak heart.

At all stages, mitochondria varied in shape. Mitochondria aggregated around the nucleus, and the shape was circular in 1-day-old yaks (Fig 2A). Elongated mitochondria scattered between the myofibrils and mitochondrial ridge, which were uniform and well distributed and were seen in 6-month-old yaks (Fig 2B). The ridge of mitochondria became irregular; concentrated and loose areas were found in mitochondria in 5-year-old yaks (Fig 2C).

**Fig 3:**
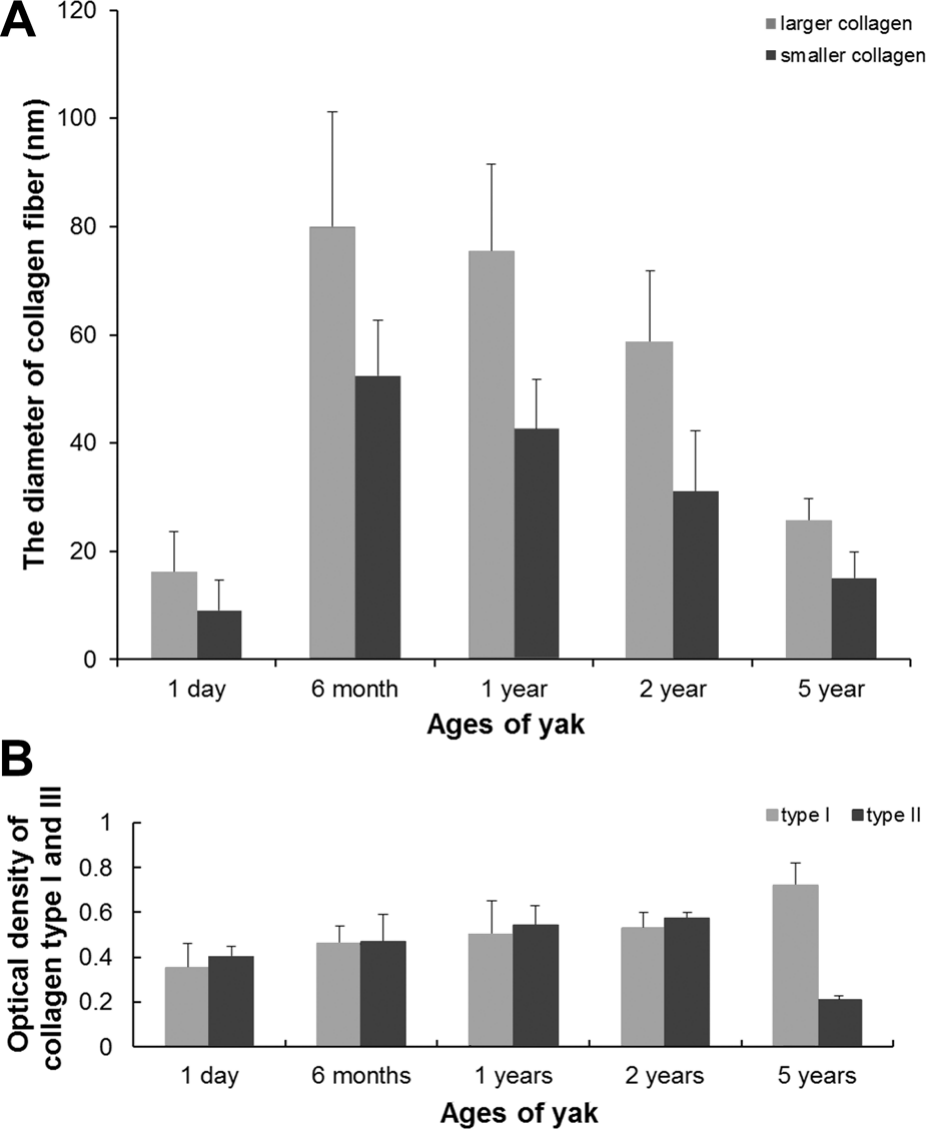
A: The diameter of collagen fiber. B: The optical density of collagen fiber.

**Fig 4:**
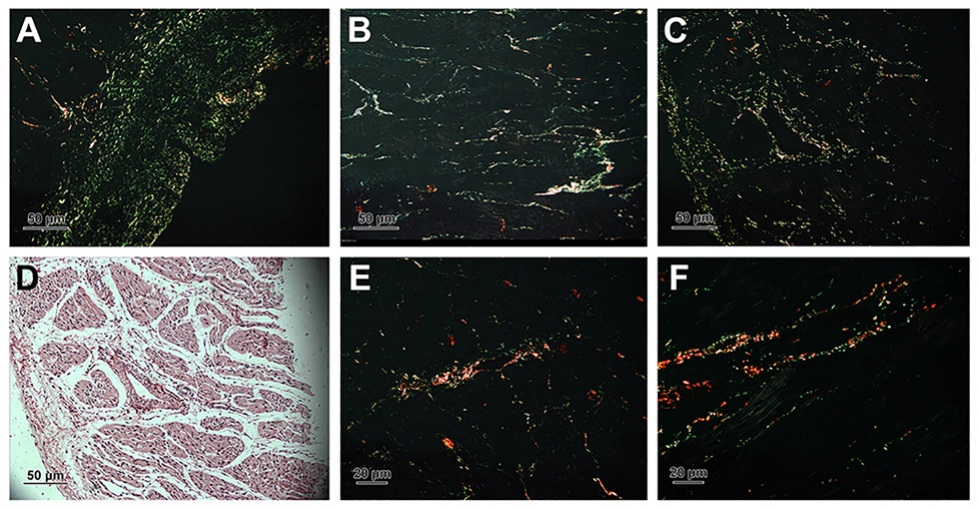
Collagen type Ⅰand Ⅲ stained in picrosirius and photographed with polarization microscope. A, B, C, E and F were the photos of collagen fiber in 1-day, 6-month, 1-year, 2-year and 5-year yak heart. D: Normal focus map of C.

Closely packed collagen fibrils were seen in the cardiac interstitium under TEM. Two types of collagens were found when their cross-sections were examined. One type of collagen fibers appeared as high-electron density, dark staining, smaller density, and smaller diameter, while the other appeared as low-electron density, pale staining, larger density, and larger diameter. The diameter of collagen in the heart of 1-day-old yaks was the smallest (Fig 2D), compared to that of 6-month-old (Fig 2E) and 2-year-old yaks (Fig 2F) (p < 0.05). The diameter of collagen fiber is seen in Fig 3A. In the heart of each ages, the perimysium presents type I (thick, yellow or red, strongly birefringent collagen fibers) and type III (thin, pale fibers of greenish color) (Fig 4). Collagen type I predominate in 5 years yak compared with other ages (p < 0.05) (Fig 3B).

### Mitochondrial density and activity changes with age

**Fig 5:**
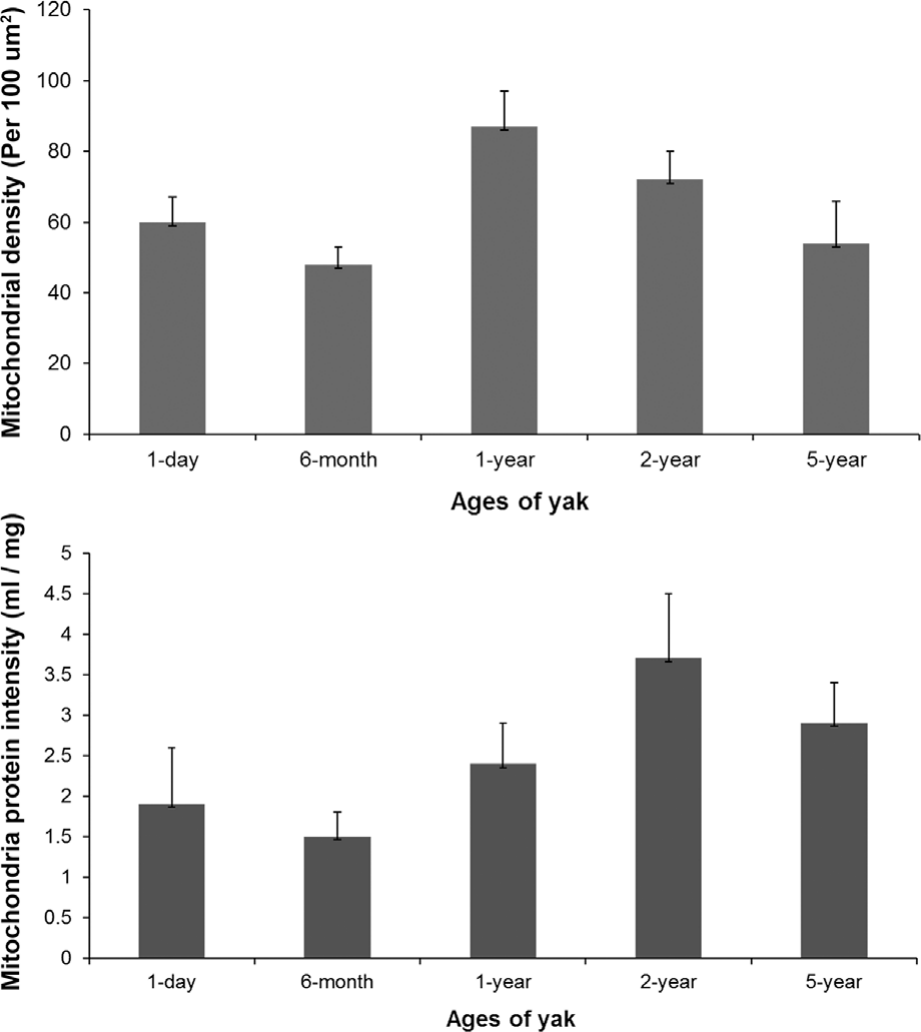
The density and protein intensity of mitochondria in the yak heart.

The number of mitochondria per 100 μm^2^ and the intensity of mitochondria protein in each age of yaks were analyzed (Fig 5). The activity of complexes I, II, and V increased with aging. However, the activity of complexes III and IV increased in yaks from the age of 1 day to 1 year, and then decreased with aging (Table 4).

**Table 4:** Enzymatic activities of Complex I-V of mitochondria. Unit: nmoles/min/mg protein.

Age	n	Complex I	Complex II	Complex III	Complex IV	Complex V
1 day	20	65.0±8.5 a	49.1±5.0a	1.20±0.1a	57.1±5.8a	70.0±10.3a
*6 month*	20	88.7±6.7b	81.4±10.3b	4.58±0.9b	191.2±17.3b	96.5±12.2a
1 year	20	91.3±9.2b	95.5±9.6b	4.46±0.7b	218.5±10.2b	148.2±47.0b
2 year	20	120.9±18.2c	141.1±21.4c	2.29±0.2c	80.1±9.0c	333.6±52.8c
5 year	20	150.1±15.5d	163.3±30.1d	1.89±0.4c	72.0±29.3c	350.9±70.3c

Note: Values are means ± SD. Different letters a, b, c and d represent that the difference was significant in the same column (p < 0.05), the same letter represent that the difference was not significant in the same column (p > 0.05).

### Protein expression and localization of HIF-1α and VEGF in the yak heart

**Fig 6:**
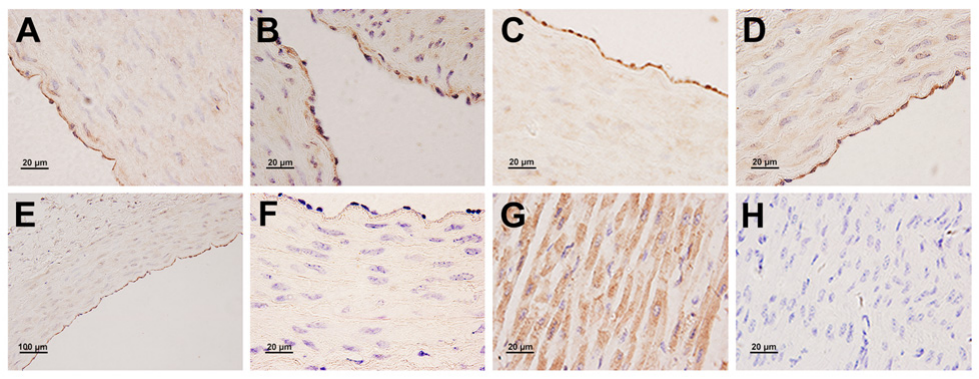
Immunohistochemical localization of HIF-1α protein in the yak heart. A, B, C, D, E: A strong positive immunostaining of HIF-1α protein in endothelial cells of coronary arterial in 1 days, 6 months, 1 year, 2 years and 5 years old yak, respectively. G: A strong positive immunostaining of HIF-1α protein in cardiac muscle cells. F, H: Control of HIF-1α expression in the coronary arterial and myocardium.

**Fig 7:**
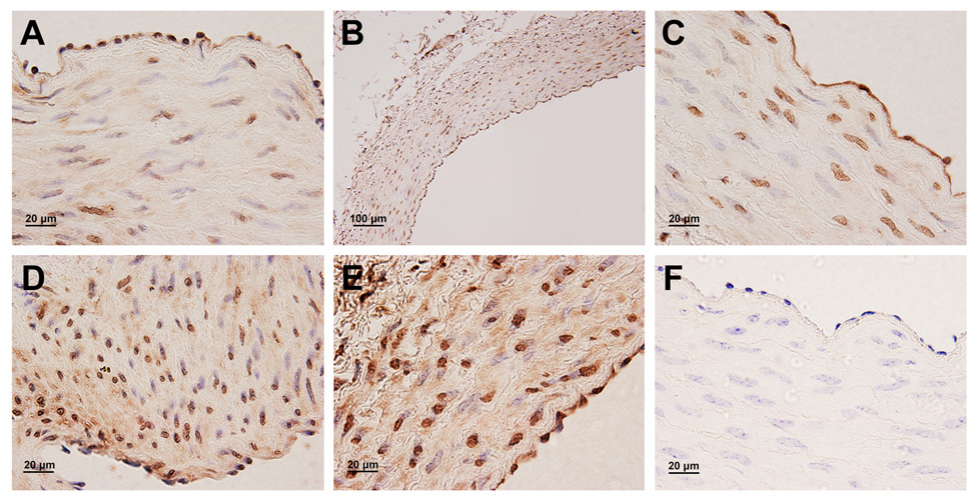
Immunohistochemical localization of VEGF protein in the yak heart. A, B, C, D, E: VEGF protein expressed in endothelial cells and smooth muscle cells of coronary artery in 1 days, 6 months, 1 year, 2 years and 5 years old yak, respectively. F: Control of VEGF expression in the coronary artery of yak.

In general, a strong positive immunostaining of HIF-1α protein was observed in the majority of endothelial cells and cardiac muscle cells (Fig 6). In contrast, no expression of VEGF protein was found in cardiac muscle cells. VEGF protein was not only observed in endothelial cells, but also in smooth muscle cells of the coronary arteries (Fig 7). In addition, the immunostaining was not observed in endothelial cells and smooth muscle cells of the coronary artery and cardiac muscle cells of the corresponding control sections (Fig 6F and 6H and Fig 7F).

**Fig 8:**
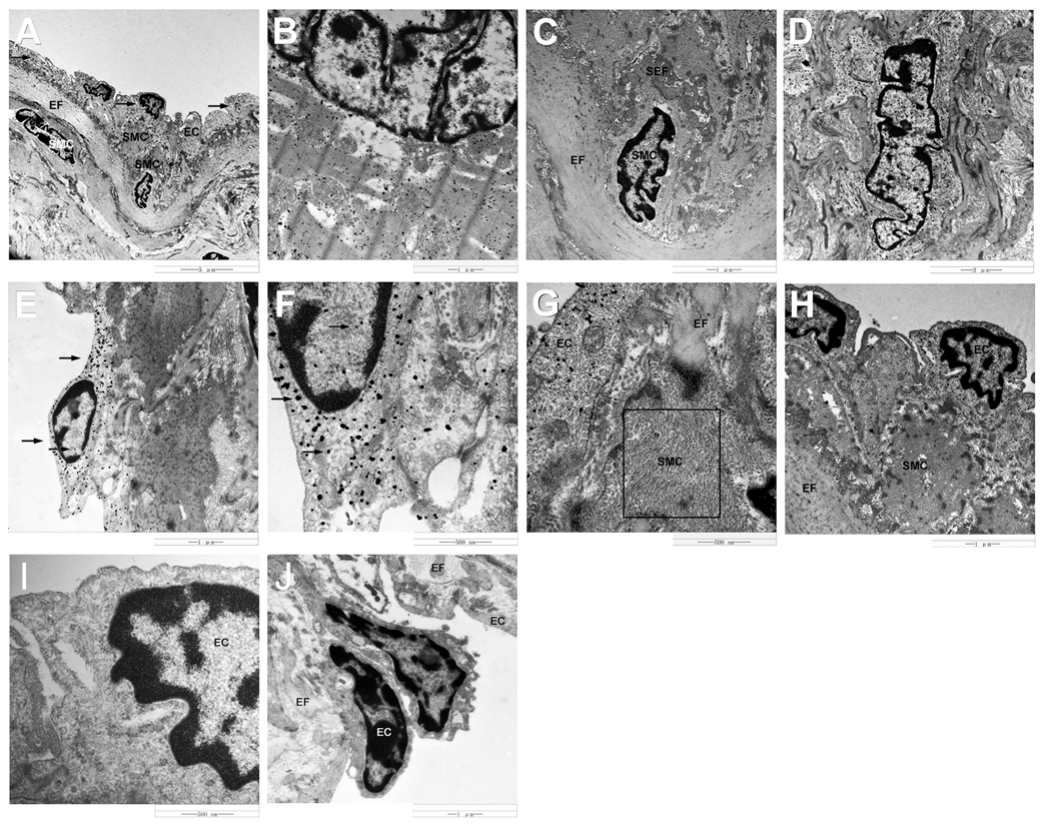
Immunogold labeling of HIF-1α and VEGF protein in the yak heart. A: Electron microscope images showing HIF-1α immunopositive silver grains in endothelial cells (arrowheads). B: HIF-1α immunopositive silver grains in cardiac muscle cells. C: No HIF-1α immunopositive silver grains in smooth muscle cells. D: Electron microscope images showing VEGF immunopositive silver grains in smooth muscle cells. E: VEGF immunopositive silver grains in endothelial cells (arrowheads). F: High magnification of VEGF immunopositive silver grains in endothelial cells (arrowheads). G: Some fine filaments were found in smooth muscle cells (boxed area). H: Smooth muscle cell has a higher electron density than endothelial cells. I, J: No gold particles were found in control sections. SMC: smooth muscle cell, EC: endothelial cell, EF: elastic fiber, SEF: secondary elastic fiber.

Immunogold labeling with silver enhancement showed the localization of HIF-1α protein on the cytoplasm (strong signals) and nuclei (weak signals) of endothelial cells (Fig 8A), and cytoplasm (strong signals) of cardiac muscle cells (Fig 8B). However, no gold particles were found in smooth muscle cells of the coronary artery localized with HIF-1α, including smooth muscle cells moved to the vascular intima (Fig 8C). In contrast, immunoelectronmicroscopy labeling showed the localization of VEGF protein in the nuclei and perinuclei of smooth muscle cells of the coronary artery (Fig 8D), and no signals presented on the cardiac muscle cells. In addition, VEGF protein was also found in the cytoplasm (strong signals) and nuclei (weak signals) of endothelial cells (Fig 8E and 8F).

Some fine filaments were found in smooth muscle cells (Fig 8G), which led the smooth muscle cell has a higher electron density than endothelial cells (Fig 8H). The phenomenon of smooth muscle cells moving to the vascular intima was observed in yaks since the age of 6 months (Fig 8C and 8H). This phenomenon becomes more common with age. However, no smooth muscle cells were found in the vascular intima in the heart of 1-day-old yaks. In addition, the gold particles were not observed in control sections (Fig 8I and 8J).

### HIF-1α and VEGF ELISA

**Fig 9:**
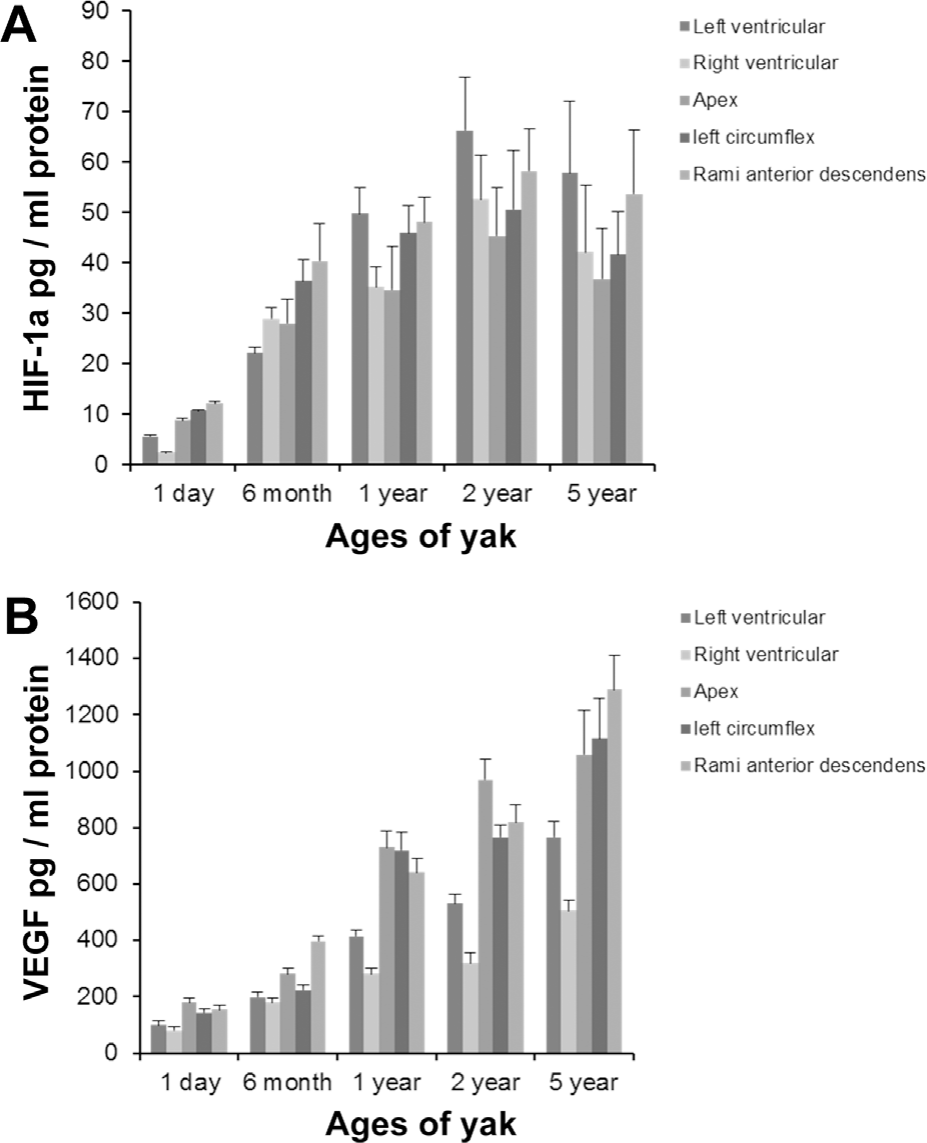
HIF-1α and VEGF protein expression in the yak heart were measured by ELISA. A: HIF-1α expression. B: VEGF expression.

The secretion of HIF-1α and VEGF from the myocardium and coronary artery was assayed by ELISA. Fig 9A showed that HIF-1α protein significantly increased in the myocardium and coronary artery from 1-day-old to 2-year-old yak heart, and decreased in the 5-year-old yak heart. A significant difference was found between yaks of 1 day and 2 years in age (p < 0.01). However, VEGF protein increased in the myocardium and coronary artery with aging (Fig 9B). The largest value of VEGF presented in 5-year-old yaks; a significant difference was found between yaks of 1 day and 5 years in age (p < 0.05). The values of HIF-1α and VEGF were bigger in the left ventricular wall than in the right ventricular wall, and the same were bigger in rami anterior descendants than in the left circumflex.

## Discussion

**Fig 10:**
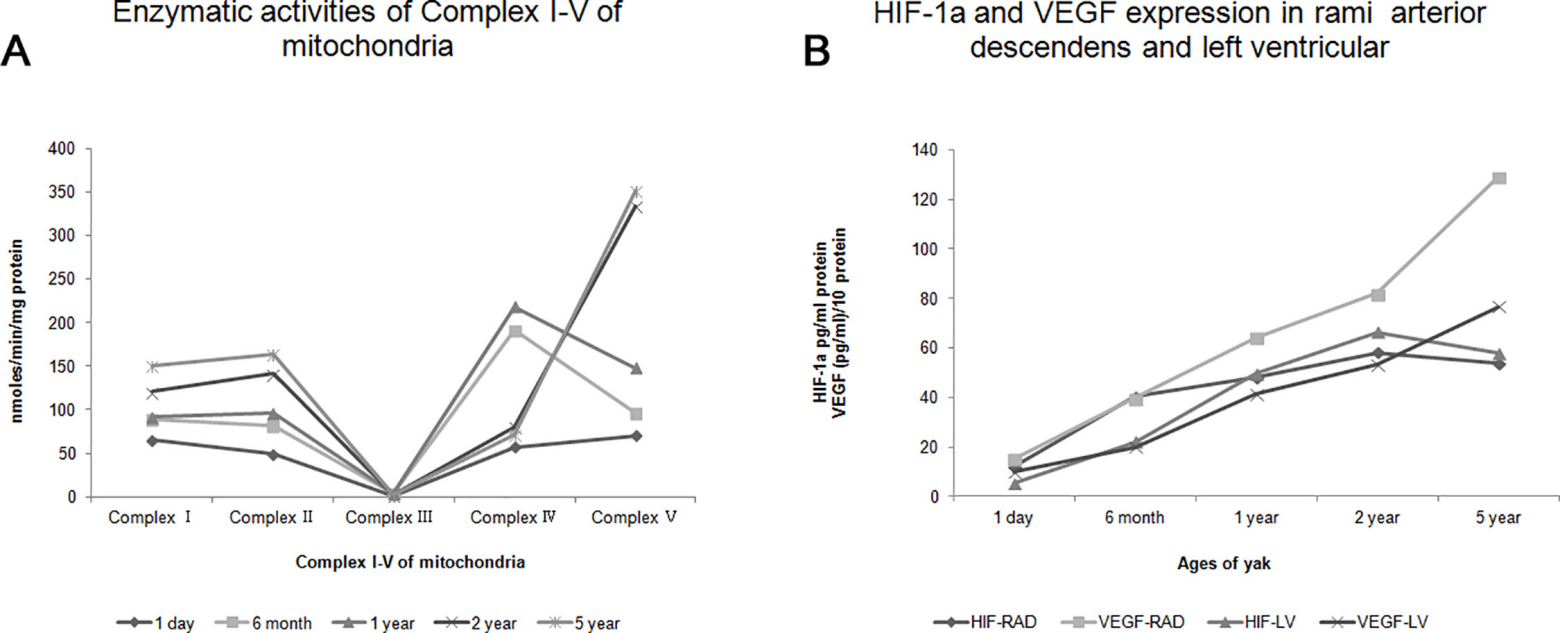
A: Enzymatic activities of Complex I-V of mitochondria of heart in each age. B: HIF-1a and VEGF expression in rami arterior descendens and left ventricular. RAD: rami arterior descendens, LV: left ventricular.

The research of yak has its particularity, the same as Tibetans and Tibetan antelope. Firstly, yak is a special and precious species lived the Tibetan highlands, there is no yak life at low altitude. Secondly, yak and cattle belongs to a different species. We did not choose cattle as a control since there are many variables to consider including species differences and altitude. We could be seen from Fig 10A that the activities of complexⅠ-Ⅴin mitochondria of 1 day old samples were the lowest, and Fig 10B that the data of 1 day old samples are very low for both HIF-1a and VEGF in rami anterior descendents and left ventricular which were dominant in the heart of yak. Therefore, as a researcher working with yaks, we argue that the best control was the newborn yak. Thirdly, the present research has proved that Tibetans, the Tibetan antelope and yak obtained genetic features of high-altitude hypoxia adaption at the physiological, morphological, and genetic levels [1,2,20,21].

In accordance with the age sequence studied, the ratio of heart weight and body weight was 1.2%, 0.96%, 0.79%, 1.2%, and 1.4%. The high ratio in the case of 1-day-old yaks may be attributed to the small birth weight, which is consistent with the findings in Tibetans [2]. However, the high ratio in the case of old yaks may be attributed to the aging of the heart. In accordance with the age sequence studied, the thickness ratio of the left and right ventricular wall in central part of heart was 1.56, 2.46, 2.56, 2.68, and 2.67, respectively. According to reports in the literature, the left ventricular wall thickness in horses, cattle and pig is 1.9, 2.33 and 2.22 times that of the right ventricular wall thickness [24]. This study demonstrated the left ventricular wall thickness also less than three times that of the right ventricular wall thickness in yaks. It can be explained that the right ventricular hypertrophy was not present in yaks. Heath et al. has proved that there is no Pulmonary hypertension in yak [25]. Zhou et al. reported that there were infact smooth muscle cells presented in the intrapulmonary arteriole of lung in yak, and the low oxygen environment can cause the thickening of the tunicae media of pulmonary artery of yak [26]. Researchers also found that the pulmonary artery of adult yak had more elastic fibers than that of adult cattle, which could maintain good retraction force of the pulmonary artery and ejecting blood [27]. Comnined these reports in lung of yaks with our findings of this study that there is no right ventricular hypertrophy in yak, we hypothesized that no right ventricular hypertrophy in yak may be due to the lack of pulmonary hypertension in the yak, and we speculated that the lack of partial pressure was compensated by increasing the elasticity of pulmonary blood vessels. In addition, the thickness of interventricular septum evidently increased with aging, and a significant difference was found between adult and old yaks (p <0.05).

The thickness of intima and tunicae media in the coronary artery increased with age, and a significant difference was found between young and adult yaks (p < 0.05). The most obvious increase was observed in rami anterior descendants. Intimal thickness is significantly higher in the coronary arteries of humans living at gigh-altitude [28,29]. The ratio of intima and tunicae media in yaks of age 1 day, 6 months, 1 year, 2 years, and 5 years were 0.05, 0.03, 0.03, 0.03, and 0.03, respectively, which indicated that this did not occur in the coronary artery of yaks. In addition, the internal elastic membrane in the coronary arteries of yaks appeared to be easily ruptured from the age of 6 months onwards. At later ages, we observed the formation of the secondary elastic membranes, which also appeared to ruputer easily in older yaks. This suggested that yaks have alteriations to their elastic membranes, which change with aging. This could explain why we observed occasional invasion of smooth muscle cells into the intima in yaks after the age of 6 months. This phenomenon of weakened internal elastic membranes and ingression of smooth muscle cells have not been reported in other reference, and could be an adaptive structure of yaks.

Stenger et al. [30] reported varied facets of the fine structures of rats and dogs at length. Brook et al. [9] observed the ultrastructure of the myocardium from early fetal life to adult life in sheep. Researchers [31,32] summarized the dominance of the areae compositae structure in mammals. Although the structures and features in many respects were analogical, there was a considerably different degree of development of specific features. An extensive research on molecular biology of cardiomyocytes [31,33,34] provideds the most basic and important information that was unavailable previously for this species.

Moore and Ruska [35] observed a spiral and concentric orientation of the cristae in mitochondria. In this study, a uniform and well-distributed ridge was found in the 6-month-old yak heart, and irregularly distributed ridge and giant mitochondria were found in the 5-year-old yak heart. Chanter et al. [36] considered that aging is associated with the extent of cardiac function changes. Preston et al. [15] found that the density and enzymatic activity of the mitochondria of heart in adult rats was higher than those of aged rats. In this study, the enzymatic activity of complexes I, II, and V increased with age; however, the highest enzymatic activity of complexes III and IV presented in the 1-year-old yak heart. It inferred that complexes III and IV have a greater relationship with adaption to hypoxia. The value of the density and intensity of mitochondria protein in cardiac muscle cells were minimum in 6-month-old yaks. It was concluded that 6 months is a key age in yaks, before which there are some adaptive changes to deal with the low-oxygen environment. In addition, the density of mitochondria decreased after yaks attain an age of 1 year, which may be because of the presence of giant mitochondria in 5-year-old yaks. Overall, it was assumed that there was a maturation of mitochondria in yaks from the age of 6 months to 2 years, in which mitochondria decreased in density and increased in protein intensity, thus making yaks to adapt better to their environment.

A number of studies have reported that pathological alterations in myocardial collagen raised probabilities of irregular pulse and impaired oxygen diffusion [37,38]. In addition, researchers revealed that the major types of collagen fibers in heart were collagens I and III [39], and there were diversity in quantity and types of collagen with aging in heart [10,40,41]. This study also found some changes in the diameter of collagen fibers. Gazoti [42] reported that the fibril diameter of human heart in the young age group was 20–70 nm and the diameter was 50–90 nm in the old age group. The findings of this study demonstrated that the diameter of collagen fiber in the 6-month-old yak heart was the largest, compared with other ages of yaks. It was not in accordance with the results in human beings, but it was a coincident with the diameter of capillary in the yak heart [24]. Based on these findings, it was hypothesized that there was decrease in the diameter and increase in the density of collagen fibers after yaks attain an age of 6 months. Olivetti [43] and Gazoti [42] suggested that the factor responsible for this change was the loss of myocytes, which cannot be substituted for other cells after death. The author of this study agreed the idea and insisted that there is a strong relationship with heart pressure-overload. These consequences could provide a theoretical virtue and an adaptive reaction to increase after load [44,45]. It is well known that the tensile strength of collagen type I is compared with that of steel, whrreas collagen type III has more distensible properties than collagen type I. We also found that collagen I increases from adult to old yak. Thus, we agree the idea of Werzar [46] that the type I collagen linkage with aging, may contribute to the decrease in the ventricular elasticity with aging. After quantified, we argued that larger diameter collagen fiber observed under TEM was collagen type I, smaller diameter collagen fiber was collagen type III. These two results were consistence.

It is known that HIF-1α pathway is activated when cells are exposed to lower oxygen tension. In normal oxygen content conditions, HIF-1α was mainly localized in the cytosol. However, this study demonstrated that HIF-1αexpressed not only in the cytosol, but also in the nucleus. This result was in accordance with the report of Dai et al. [13] who demonstrated that the translocation of HIF-1α into the nucleus is induced by hypoxia, and it is also consistent with similar findings in the pulmonary vascular [47]. It was also found in this study that the levels of expression of HIF-1α elevated significantly in yaks since the age of 1 day, increased gradually during the growth stage of yaks since the age of 6 months, and decreased with age, suggesting a vital effect of HIF-1α in the growth of yaks under hypoxia. Authors of this study insisted that chronic hypoxia and development of heart increased the expression of HIF-1α. HIF-1α played an active function in the physiological adaption in yak. VEGF is one of the downstream genes of HIF-1α. VEGF was found mainly in endothelial cells and smooth muscle cells of the coronary artery, suggesting a key role in vascular remolding in the heart. Tang et al. [48] reported that the elevated expression was accompanied by increased vascular density. This study demonstrated that the VEGF release increased gradually from the age of 1 day to 5 years. Combined with our previous research conclusion of cardiac capillary bed of yaks, which proved that the capillary diameter decreased and the density of capillary increased with aging [21], we insisted that both results have the promising consistency. It inferred that VEGF has a positive role during the development of yak heart. In addition, the changes of HIF-1α expression was parallel to that of the VEGF expression, and the secretion value of VEGF was bigger than that of HIF-1α, suggesting that HIF-1α regulated the VEGF expression in the yak heart.

## Conclusions

The following conclusions can be drawn from this study: (1) the phenomenon of no right ventricular hypertrophy was a manifestationof adaptive structure of yaks with aging. (2) HIF-1α and VEGF protein played an active role in physiological adaption in response to hypoxia and development of heart in yak. (3) Yak heart adapts from 1 day to hypoxia. From birth to 6 months self-adaption happens mainly, from 6 months to 1 year both self-adaption and growing process occurred, from 1 year to 2 years, there is a maturation of yak heart making it better adaption.

## References

[1] Ge RL, Cai Q, Shen Y Y, San A, Ma L, Zhang Y, et al Draft genome sequence of the Tibetan antelope. Nat Commun. 2013;4: 1858 10.1038/ncomms2860 23673643 PMC3674232

[2] Simonson TS, Yang Y, Huff CD, Yun H, Qin G, Witherspoon DJ, et al Genetic evidence for high-altitude adaptation in Tibet. Science. 2010;329: 72–75. 10.1126/science.1189406 20466884

[3] Alexander A, Jensen R. Gross cardiac changes in cattle with high mountain (brisket) disease and in experimental cattle maintained at high altitudes. American Journal of Veterinary Research. 1959;20: 680–689.

[4] Recavarren S, Arias-Stella J. RIGHT VENTRICULAR HYPERTROPHY IN PEOPLE BORN AND LIVING AT HIGH ALTITUDES. Br Heart J. 1964;26: 806–812. 14222550 10.1136/hrt.26.6.806 PMC1018133

[5] Crick SJ, Sheppard MN, Ho SY, Gebstein L, Anderson RH. Anatomy of the pig heart: comparisons with normal human cardiac structure. J Anat. 1998;193 (Pt 1): 105–119. 9758141 10.1046/j.1469-7580.1998.19310105.x PMC1467827

[6] Macdonald AA, Carr PA, Currie RJ. Comparative anatomy of the foramen ovale in the hearts of cetaceans. J Anat. 2007;211: 64–77. 10.1111/j.1469-7580.2007.00743.x 17532800 PMC2375797

[7] Sakabe M, Abe M, Nakatani K, Ikeda K, Yoshiyamal M, Nakajima Y. [Dissection of the heart: a useful guide to understanding the three-dimensional gross anatomy]. Kaibogaku Zasshi. 2006;81: 117–124. 17191690

[8] Wessels A, Sedmera D. Developmental anatomy of the heart: a tale of mice and man. Physiol Genomics. 2003;15: 165–176. 10.1152/physiolgenomics.00033.2003 14612588

[9] Brook WH, Connell S, Cannata J, Maloney JE, Walker AM. Ultrastructure of the myocardium during development from early fetal life to adult life in sheep. J Anat. 1983;137 (Pt 4): 729–741. 6668250 PMC1171875

[10] de Souza RR. Aging of myocardial collagen. Biogerontology. 2002;3: 325–335. 12510171 10.1023/a:1021312027486

[11] Shao SX, Zhang L, Duan XL, Lu P, Li JB. Ultrastructure changes of human fetal myocytes in different months. Progress of Anatomical Sciences 2001;1: 30–32.

[12] Shimada T, Zhang L, Takeshita M. Ultrastructural differences between the working myocardial cells and the conduction system in the monkey heart. Medical Electron Microscopy. 1994;27: 227–231.

[13] Dai Y, Xu M, Wang Y, Pasha Z, Li T, Ashraf M. HIF-1alpha induced-VEGF overexpression in bone marrow stem cells protects cardiomyocytes against ischemia. J Mol Cell Cardiol. 2007;42: 1036–1044. 10.1016/j.yjmcc.2007.04.001 17498737 PMC1995444

[14] Eleftheriadis SG, Sivridis E, Koutsopoulos A, Galatoudis ZG, Chloropoulou PA, Koukourakis MI, et al One-lung ventilation and HIF1alpha expression in lung cancer and pneumothorax. Anticancer Res. 2010;30: 1143–1148. 20530420

[15] Tipoe GL, Fung ML. Expression of HIF-1alpha, VEGF and VEGF receptors in the carotid body of chronically hypoxic rat. Respir Physiol Neurobiol. 2003;138: 143–154. 14609506 10.1016/s1569-9048(03)00188-5

[16] Yu AY, Shimoda LA, Iyer NV, Huso DL, Sun X, McWilliams R, et al Impaired physiological responses to chronic hypoxia in mice partially deficient for hypoxia-inducible factor 1alpha. J Clin Invest. 1999;103: 691–696. 10.1172/jci5912 10074486 PMC408131

[17] Markel TA, Wang Y, Herrmann JL, Crisostomo PR, Wang M, Novotny NM, et al VEGF is critical for stem cell-mediated cardioprotection and a crucial paracrine factor for defining the age threshold in adult and neonatal stem cell function. Am J Physiol Heart Circ Physiol. 2008;295: H2308–2314. 10.1152/ajpheart.00565.2008 18849336 PMC2614539

[18] Lin F, Pan LH, Ruan L, Qian W, Liang R, Ge WY, et al Differential expression of HIF-1alpha, AQP-1, and VEGF under acute hypoxic conditions in the non-ventilated lung of a one-lung ventilation rat model. Life Sci. 2015;124: 50–55. 10.1016/j.lfs.2014.12.020 25592135

[19] Poynter JA, Manukyan MC, Wang Y, Brewster BD, Herrmann JL, Weil BR, et al Systemic pretreatment with dimethyloxalylglycine increases myocardial HIF-1alpha and VEGF production and improves functional recovery after acute ischemia/reperfusion. Surgery. 2011;150: 278–283. 10.1016/j.surg.2011.06.006 21801965

[20] Duan D, Yu S, Cui Y. Morphological study of the sinus node and its artery in yak. Anat Rec (Hoboken). 2012;295: 2045–2056. 10.1002/ar.22591 23044916

[21] He YY, Yu SJ, Cui Y, Du P. Morphological study on microvasculature of left ventricular wall in infant and adult yaks. Anat Rec (Hoboken). 2010;293: 1519–1526. 10.1002/ar.21201 20652942

[22] Junqueira LC, Cossermelli W, Brentani R. Differential staining of collagens type I, II and III by Sirius Red and polarization microscopy. Arch Histol Jpn. 1978;41: 267–274. 82432 10.1679/aohc1950.41.267

[23] Junqueira LC, Bignolas G, Brentani RR. Picrosirius staining plus polarization microscopy, a specific method for collagen detection in tissue sections. Histochem J. 1979;11: 447–455. 91593 10.1007/BF01002772

[24] Li LY, Xu JH. Anatomy of domestic animal. ed. Taiwan; 1966.

[25] Heath D, Williams D, Dickinson J. The pulmonary arteries of the yak. Cardiovasc Res. 1984;18: 133–139. 6705004 10.1093/cvr/18.3.133

[26] Zhou JX, Yu SJ, He JF, Cui Y. Segmentation features and structural organization of the intrapulmonary artery of the yak. the Anat Rec (Hoboken). 2013;296: 1775–1788. 24123963 10.1002/ar.22790

[27] Zhou JX, Yu SJ, He JF, Cui Y. Comparative structural organization and morphological analyses of intrapulmonary artery of adult yak and cattle. Chin J Vet Sci. 2015;35: 1840–1862.

[28] Sims FH, Gavin JB, Vanderwee MA. The intima of human coronary arteries. Am Heart J. 1989;118: 32–38. 2741794 10.1016/0002-8703(89)90068-9

[29] Sims FH, Gavin JB. The early development of intimal thickening of human coronary arteries. Coronary Artery Disease. 1990;1: 205–214.

[30] Stenger RJ, Spiro D. The ultrastructure of mammalian cardiac muscle. J Biophys Biochem Cytol. 1961;9: 325–351. 19866581 10.1083/jcb.9.2.325 PMC2225002

[31] Franke WW, Borrmann CM, Grund C, Pieperhoff S. The area composita of adhering junctions connecting heart muscle cells of vertebrates. I. Molecular definition in intercalated disks of cardiomyocytes by immunoelectron microscopy of desmosomal proteins. Eur J Cell Biol. 2006;85: 69–82. 10.1016/j.ejcb.2005.11.003 16406610

[32] Pieperhoff S, Franke WW. The area composita of adhering junctions connecting heart muscle cells of vertebrates. VI. Different precursor structures in non-mammalian species. Eur J Cell Biol. 2008;87: 413–430. 10.1016/j.ejcb.2008.02.005 18420304

[33] Barker RJ, Price RL, Gourdie RG. Increased association of ZO-1 with connexin43 during remodeling of cardiac gap junctions. Circ Res. 2002;90: 317–324. 11861421 10.1161/hh0302.104471

[34] Lubkemeier I, Requardt RP, Lin X, Sasse P, Andrie R, Schrickel JW, et al Deletion of the last five C-terminal amino acid residues of connexin43 leads to lethal ventricular arrhythmias in mice without affecting coupling via gap junction channels. Basic Res Cardiol. 2013;108: 348 10.1007/s00395-013-0348-y 23558439 PMC3678986

[35] Moore DH, Ruska H. Electron microscope study of mammalian cardiac muscle cells. J Biophys Biochem Cytol. 1957;3: 261–268. 13438909 10.1083/jcb.3.2.261 PMC2224083

[36] Chantler PD, Goldspink DF, Clements RE, Sharp L, Schlosshan D, Tan LB. Congestive heart failure: extent of cardiac functional changes caused by aging and organ dysfunction. Heart. 2006;92: 686–688. 10.1136/hrt.2005.063578 16614287 PMC1860956

[37] Lopez Salazar B, Ravassa Albeniz S, Arias Guedon T, Gonzalez Miqueo A, Querejeta R, Diez Martinez J. Altered fibrillar collagen metabolism in hypertensive heart failure. Current understanding and future prospects. Rev Esp Cardiol. 2006;59: 1047–1057. 17125715 10.1157/13093982

[38] Vasan RS, Benjamin EJ. Diastolic heart failure—no time to relax. N Engl J Med. 2001;344: 56–59. 10.1056/nejm200101043440111 11136963

[39] Collier P, Watson CJ, van Es MH, Phelan D, McGorrian C, Tolan M, et al Getting to the heart of cardiac remodeling; how collagen subtypes may contribute to phenotype. J Mol Cell Cardiol. 2012;52: 148–153. 10.1016/j.yjmcc.2011.10.002 22008391

[40] Eghbali M, Eghbali M, Robinson TF, Seifter S, Blumenfeld OO. Collagen accumulation in heart ventricles as a function of growth and aging. Cardiovasc Res. 1989;23: 723–729. 2598224 10.1093/cvr/23.8.723

[41] Medugorac I. Collagen content in different areas of normal and hypertrophied rat myocardium. Cardiovasc Res. 1980;14: 551–554. 6452208 10.1093/cvr/14.9.551

[42] Gazoti Debessa CR, Mesiano Maifrino LB, Rodrigues de Souza R. Age related changes of the collagen network of the human heart. Mech Ageing Dev. 2001;122: 1049–1058. 11389923 10.1016/s0047-6374(01)00238-x

[43] Olivetti G, Melissari M, Capasso JM, Anversa P. Cardiomyopathy of the aging human heart. Myocyte loss and reactive cellular hypertrophy. Circ Res. 1991;68: 1560–1568. 2036710 10.1161/01.res.68.6.1560

[44] Kasner M, Westermann D, Lopez B, Gaub R, Escher F, Kuhl U, et al Diastolic tissue Doppler indexes correlate with the degree of collagen expression and cross-linking in heart failure and normal ejection fraction. J Am Coll Cardiol. 2011;57: 977–985. 10.1016/j.jacc.2010.10.024 21329845

[45] Valencia Serrano F, López Salazar B, Gomez Doblas J, Rodriguez Bailon I, Porras C, Melero J, et al (2007) Non-invasive assessment of myocardial fibrosis in severe aortic stenosis patients. European Heart Journal. Oxford Univ Press Great Clarendon St, Oxford Ox2 6dp, ENGLAND. pp. 653–653.17329408

[46] Verzar F. Factors which influence the age-reaction of collagen in the skin. Gerontologia. 1964;43: 209–221. 14276057

[47] HE J-f, CUI Y. Distribution of vascular endothelial growth factor in plateau yak's lung. Chinese Veterinary Science. 2008;12: 015.

[48] Tang YL, Zhao Q, Zhang YC, Cheng L, Liu M, Shi J, et al Autologous mesenchymal stem cell transplantation induce VEGF and neovascularization in ischemic myocardium. Regul Pept. 2004;117: 3–10. 14687695 10.1016/j.regpep.2003.09.005

